# Training flexible conceptual retrieval in stroke aphasia

**DOI:** 10.1080/09602011.2021.1895847

**Published:** 2021-03-14

**Authors:** Sara Stampacchia, Glyn P. Hallam, Hannah E. Thompson, Upasana Nathaniel, Lucilla Lanzoni, Jonathan Smallwood, Matthew A. Lambon Ralph, Elizabeth Jefferies

**Affiliations:** 1 Department of Psychology and York Neuroimaging Centre, University of York, York, YO10 5DD, UK; 2 Laboratory of Neuroimaging and Innovative Molecular Tracers (NIMTlab), Geneva University Neurocenter and Faculty of Medicine, University of Geneva, Geneva, Switzerland; 3 Department of Psychology, School of Human and Health Sciences, University of Huddersfield, Huddersfield, HD1 3DH; 4 School of Psychology, University of Surrey, Guildford, GU2 7XH; 5 Psychology Department, University of Haifa, Israel; 6 Queen's University, Kingston, Ontario, Canada; 7 Cognition and Brain Sciences Unit, Chaucer Road, University of Cambridge, Cambridge, UK

**Keywords:** aphasia, semantic, training, cognitive control, executive

## Abstract

Semantic therapy in post-stroke aphasia typically focusses on strengthening links between conceptual representations and their lexical-articulatory forms to aid word retrieval. However, research has shown that semantic deficits in this group can affect both verbal and non-verbal tasks, particularly in patients with deregulated retrieval as opposed to degraded knowledge. This study therefore aimed to facilitate semantic cognition in a sample of such patients with post-stroke semantic aphasia (SA) by training the identification of both strong and weak semantic associations and providing explicit pictorial feedback that demonstrated both common and more unusual ways of linking concepts together. We assessed the effects of this training on (i) trained and untrained items; and (ii) trained and untrained tasks in eleven individuals with SA. In the training task, the SA group showed improvement with practice, particularly for trained items. A similar untrained task using pictorial stimuli (Camel and Cactus Test) also improved. Together, these results suggest that semantic training can be beneficial in patients with SA and may show some degree of generalisation to untrained situations. Future research should seek to understand which patients are most likely to benefit from this type of training.

## 1 Introduction

Research has shown that semantic deficits arise in at least three ways–there can be difficulty activating heteromodal concepts from specific input modalities, degradation of heteromodal knowledge itself, and impairment of control processes that support access to non-dominant aspects of knowledge. These different deficits might benefit from different types of intervention. The first pattern is seen in patients with post-stroke aphasia who have difficulty accessing conceptual meaning from language, yet good understanding of pictures – as in pure word deafness and Wernicke’s aphasia (Robson, Sage, & Lambon Ralph, 2012; Thompson, Robson, Lambon Ralph, & Jefferies, 2015). Understanding the meaning of visual objects can also be specifically disrupted after posterior cerebral artery infarcts (Roberts et al., 2013). These patients are likely to benefit from compensatory strategies maximising their use of preserved input pathways.

Degradation of heteromodal concepts, in contrast, results in multimodal semantic impairment, affecting both verbal and non-verbal stimuli. Atrophy of the ventrolateral anterior temporal lobes (seen in semantic dementia, SD) leads to progressive degradation of semantic knowledge. SD patients show loss of specific and less familiar items first and consistent performance across different tasks probing the same concepts (Jefferies and Lambon Ralph, 2006; Mayberry *et al*., 2011). These patients show some benefits in training studies focussed on relearning conceptual distinctions as long as the training is continued, potentially reflecting the fact that the anterior temporal lobes (ATLs) can support patterns of relearning despite degradation (Bier et al., 2009; Heredia, Sage, Lambon Ralph, & Berthier, 2009; Hoffman, Clarke, Jones, & Noonan, 2015; Jokel et al., 2006; 2010; Mayberry, Sage, Ehsan, & Lambon Ralph, 2011; Reilly et al., 2010; Savage, Ballard, Piguet, & Hodges, 2013).

Heteromodal semantic impairment does not always reflect degraded knowledge, however. Work by our group and others (Jefferies, 2013; Jefferies & Lambon Ralph, 2006; Lambon Ralph, Jefferies, Patterson, & Rogers, 2017; Thompson-Schill, 2003) shows that semantic deficits following left-hemisphere stroke can also reflect difficulty constraining retrieval such that it is appropriate to the context or task. We have referred to this pattern as “semantic aphasia” (SA), since it affects both verbal and non-verbal manipulations of semantic knowledge, including picture matching and object use (Corbett, Jefferies, Ehsan, & Lambon Ralph, 2009; Corbett, Jefferies, & Lambon Ralph, 2011, 2009). SA patients are thought to have impaired executive control processes, which ‘shape’ conceptual retrieval following damage to left inferior frontal and/or posterior temporal regions, in the face of intact conceptual knowledge and brain damage that spares ventrolateral ATL. This causes greater impairment when non-dominant information needs to be retrieved, or when strong distractors need inhibiting (cf. Badre and Wagner, 2005; Whitney *et al*., 2011). There has been little attempt to design training or rehabilitation strategies for these patients based on this theoretical framework, although we might expect that approaches that provide practice in retrieving a range of different kinds of association (including non-dominant aspects of knowledge) might be most successful in promoting flexible patterns of semantic cognition.

Many studies employing training tasks in post-stroke aphasia have focussed on picture naming (Kiran & Bassetto, 2008) as opposed to cognitive or semantic control. Studies employing picture naming tasks tend to show a clear benefit for items that are trained multiple times, but weak generalisation to untrained items (Davis & Pring, 1991; Marshall, Pound, White-thomson, & Pring, 1990; Pring, Hamilton, Harwood, & Macbride, 1993). This suggests that such training strengthens lexical-articulatory forms, or the links between these representations and conceptual features that are activated by the picture. Semantic approaches to aphasia therapy also typically target speech production, but seek to drive improvements through greater accessibility of semantic features which converge on the target concept, allowing activation of the required lexical item (for a review, see Efstratiadou et al., 2018). In Semantic Feature Analysis (SFA), devised by Ylvisaker and Szekeres (1985), clients are asked to generate (or in some variants, verify) semantic features for concrete nouns, including superordinate category membership, properties such as colour or shape, actions, locations and associations. Meta-analyses and case-series studies suggest that SFA is generally successful at cueing lexical retrieval in picture naming tasks in post-stroke aphasia (e.g., Boyle 2010; Maddy, Capilouto & McComas, 2014): for example, Efstratiadou et al. (2018) performed a meta-analysis of 21 studies and 55 participants and found improvements in naming in 45 individuals (with 40% of the sample showing generalisation to untrained items). SFA activates the central or dominant features and associations of each item, and consequently, while it can facilitate picture naming, this approach may not be optimal for improving comprehension in SA, since these patients are able to retrieve semantic information in dominant contexts, but show reduced flexibility when weak or subordinate knowledge is required to suit the current goal or context. A related approach, Verb Network Strengthening Treatment or VNeST (Edmonds et al., 2009), involves activating when/where/why information, together with agents and recipients for verbs. There is some evidence that vNeST can produce improvements in sentence production that generalise to untrained items (e.g., Edmonds et al., 2009; Edmonds, Mammino & Ojeda, 2014) – although again, effects on semantic tasks have not been widely explored, and this approach is not aimed at the retrieval of non-dominant information.

Efforts to support semantic processing in post-stoke aphasia have been largely unsuccessful in people with poor comprehension at the single item level (e.g., Van Hees *et al*., 2013) and fewer investigations have attempted to ameliorate these comprehension problems – perhaps because it has been noted previously that semantic deficits in aphasia are often accompanied by broader deficits of cognitive control (Purdy, 2002; Jefferies and Lambon Ralph, 2006; Baldo *et al*., 2015; Thompson *et al*., 2018). Executive control is often impaired in people with post-stroke aphasia (especially in those with more significant impairment, Glosser & Goodglass, 1990; Purdy, 2002) and its preservation is thought to be necessary for strong recovery of language after stroke (Geranmayeh, Chau, Wise, Leech, & Hampshire, 2017). People with poor cognitive control respond less well to conventional speech and language therapy (Fillingham, Sage and Lambon Ralph, 2005; Fillingham, Sage and Lambon Ralph, 2005; Fillingham, Sage and Ralph, 2005; Fillingham *et al*., 2006; for a systematic literature review see Simic *et al*., 2017). This might be because such individuals are less good at allocating and maintaining attention to the training task, and/or because their primary difficulty is not weakness in any specific type of language or conceptual representation that can be overcome through practice. In fact, massed practice at retrieving the *same* specific meanings or associations would be arguably unhelpful in people with deregulated semantic cognition who have SA, since their primary problem appears to be the flexible retrieval of diverse information pertaining to the same concept at different times, depending on the context. A more successful approach might involve helping patients to access a wide range of different associations, some relatively strong and some weaker, depending on the semantic decision to be made.

The capacity to control mental activity in a flexible fashion, to suit the changing demands of a task, is highly relevant to communication and comprehension. For example, it can be necessary to focus on the subordinate meanings of ambiguous words or on specific task-relevant meanings in the context of strong distracting information. Semantic control areas – most notably left interior frontal gyrus (LIFG) and left posterior middle temporal gyrus – are activated in healthy participants by a range of semantic control manipulations, including the contrast of hard and easy semantic judgements (Badre, Poldrack, Paré-Blagoev, Insler, & Wagner, 2005; Badre & Wagner, 2007; Noonan, Jefferies, Visser, & Lambon Ralph, 2013; Thompson-Schill, D’Esposito, Aguirre, & Farah, 1997). This semantic control network partially overlaps with ‘multiple demand cortex’ that supports cognitive control across tasks (Davey et al., 2016; Duncan, 2010). Patients with SA, who have damage focused on the left-lateralised semantic control network, have particular problems accessing non-dominant semantic features and associations, and typically also have executive deficits on non-semantic tasks (Noonan, Jefferies, Corbett, & Lambon Ralph, 2010). In a recent study, we found SA patients with lesions to LIFG showed increased recruitment of undamaged nodes within the semantic control network during auditory presentation of ambiguous sentences (Hallam et al., 2017); this pattern is consistent with functional compensation in non-lesioned parts of this network. Similarly, another study found that the fMRI response to language stimuli in aphasia resembles the response evoked by hard-to-comprehend material in healthy controls (Brownsett et al., 2014), while the ability to activate cognitive control regions is predictive of recovery (Geranmayeh, Brownsett, & Wise, 2014; Geranmayeh et al., 2017). Evidence from traumatic brain injury patients suggest that cognitive control training is more effective than knowledge-based training (Vas et al., 2016) and promotes increased connectivity in multi-demand control regions (Han, Chapman, & Krawczyk, 2018). Given these observations, cognitive training might benefit people with post-stroke aphasia if it can strengthen engagement of control mechanisms within language and semantic tasks.

In this study, we trained the retrieval of diverse types of association to improve comprehension in patients with SA. Although we examined SA patients in this study, our approach might be applicable to any groups with deregulated semantic retrieval, in which heteromodal comprehension is impaired as a consequence of poor control (such as patients with lesions in semantic control key areas following non-stroke aetiologies). Volunteers were asked to decide which word was associated with a probe word, and the associations to be retrieved ranged in their associative strength from weak to strong. On each trial, the participants were helped to understand the relevant association through the provision of feedback and a linking photograph that captured the relevant association in a concrete way. We presented novel training items within each session, to encourage flexibility, but a subset of the items was also repeated across time points. In this way, we could look at the extent to which any training effect generalised to untrained items.

## 2 Participants

Eleven patients [7 females, mean age = 61.1 (SD = 11.3); mean education leaving age = 16.5 years (SD = 1.35); mean years since CVA = 7.9 (SD = 5.32)] with chronic stroke aphasia from left-hemisphere CVA were recruited from stroke and communication support groups in Yorkshire, UK. Demographic details are reported in Table 1. Patients were selected to show multimodal semantic control impairment (see section 2.1). Besides their multimodal semantic impairment, the patients had a range of other language impairments (e.g., deficits in repetition and fluency of speech), although their comprehension problems could not be entirely accounted for in these terms. None of the patients were undergoing a structured course of individual or group therapy for treatment of comprehension deficits during the course of the study, though one patient (MB) was using React2, a computerised self-guided naming therapy. This participant had been using React2 regularly for many years, making it unlikely that changes over the course of our two-week training could be attributed to React2.

### 2.1 Inclusion criteria

In line with the original use of the term “semantic aphasia” by Henry Head (1926) and the inclusion criteria proposed by Jefferies & Lambon Ralph, (2006), the patients in this study were selected to show deficits affecting the appropriate use of concepts presented as words and objects when control demands were high. In addition to verbal semantic problems, they were impaired on at least one non-verbal task (see section 4.3). Sample size was determined by the maximum number of patients available to take part in the study. These criteria for including participants were established prior to data collection. There were no other inclusion/exclusion criteria. In common with previous SA samples (e.g. Jefferies and Lambon Ralph, 2006; Stampacchia *et al*., 2018), the patients showed strong effects of semantic control manipulations across tasks (details below). Individual patient data and task descriptions are provided in section 4.3.

### 2.2 Lesion analysis

**Figure 1:**
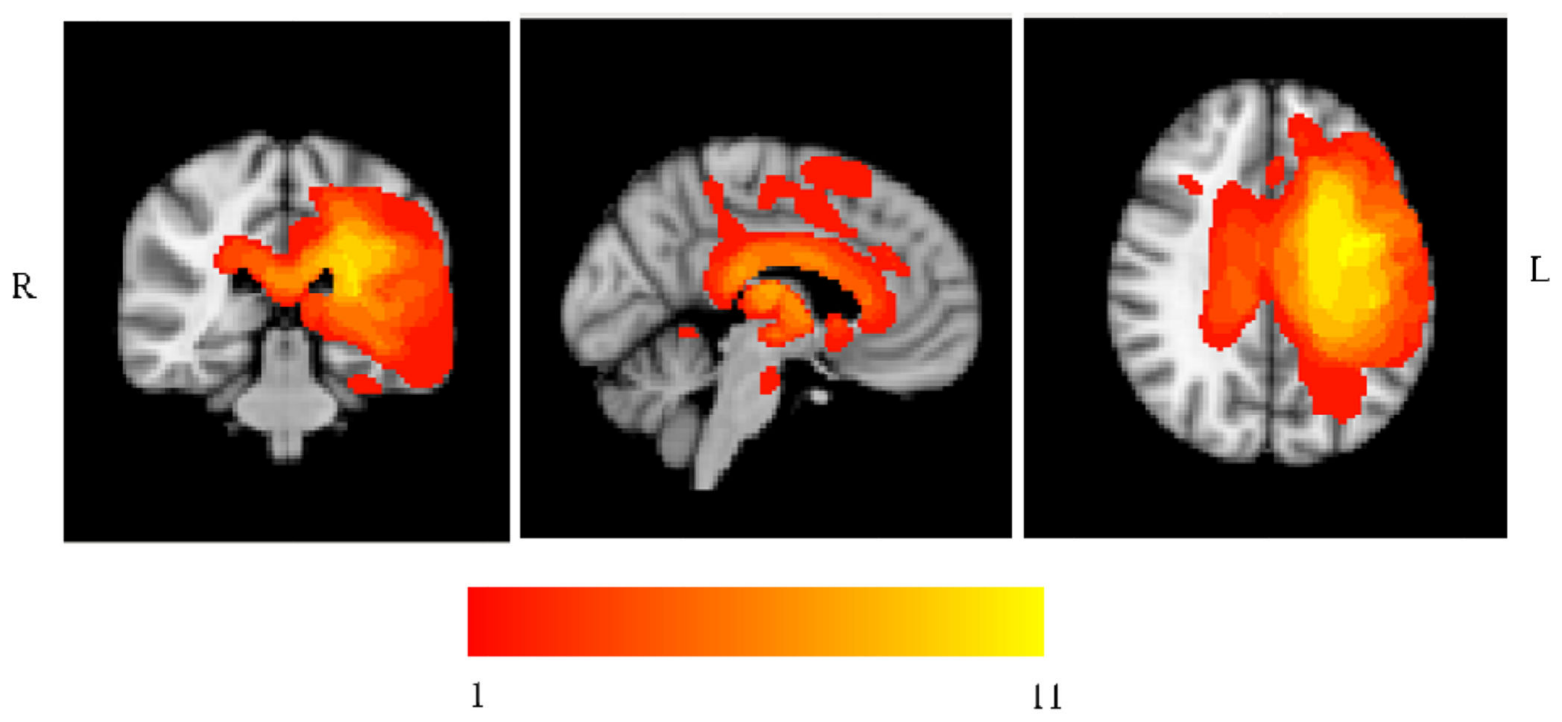
Patients’ Lesion Overlay. Lesion overlay of the sample of SA patients included in the study. Patients’ brains compared to aged-matched controls. Grey matter, white matter and CSF were segmented and changes from the healthy control brains were highlighted as ‘lesion’ using automated methods (Seghier, Ramlackhansingh, Crinion, Leff, & Price, 2008). Colour bar indicates amount of overlap from 1 to 11 patients.

MRI scans were traced onto standardized templates (Damasio & Damasio, 1989) and lesion identification was manually performed (see Table 2 and Figure 1 for lesion overlay). All eleven patients had lesions affecting left posterior LIFG; in eight cases this damage extended to mid-to-anterior LIFG. Parietal regions (supramarginal gyrus and/or angular gyrus) were also affected in 9 cases out of 11, and pMTG was affected in all but four cases. While there was some damage to ATL in 4 patients (SD, KQ, KA,VN), the ventral portion of ATL, which has been implicated in conceptual representation across modalities (Binney, Parker, & Lambon Ralph, 2012; Visser et al., 2012), was intact in all cases. This region is supplied by both the anterior temporal cortical artery of the middle cerebral artery and the anterior temporal branch of the distal posterior cerebral artery, reducing its vulnerability to stroke (Borden, 2006; Conn, 2008; Phan, Donnan, Wright, & Reutens, 2005). The hippocampus and parahippocampal gyrus were intact in all patients.

## 3 Open access and declarations

The conditions of our ethical approval do not permit public archiving of brain data, because participants did not provide sufficient consent. Researchers who wish to access the data should contact the Research Ethics Committee of the Department of Psychology, University of York, or the corresponding author. Sufficient data to replicate all results reported in the paper will be released to researchers, subject to the approval of the Research Ethics and Committee of the Department of Psychology, University of York, when this is possible under the terms of the GDPR (General Data Protection Regulation EU 2016/679). Behavioural data are provided in Open Science Framework (https://osf.io/2vuhk). Digital study materials (i.e. experimental scripts and pictorial stimuli as described in the following sections) are provided on Open Science Framework (https://osf.io/4ebgr/). The background neuropsychological materials are not provided on OSF since these included published and copyrighted tests, and because they were administrated as ’paper and pencil tests’. Researchers who wish to access these materials should contact the corresponding author. Codes of analyses (https://osf.io/gh9qz) of behavioural data are provided on Open Science Framework.

No part of the study procedures and analyses was pre-registered prior to the research being conducted. All manipulations and measures of this study are reported in the following sections.

## 4 Background neuropsychological assessment

### 4.1 Non-semantic tests

Individual scores are reported in Table 1. To characterise language processing, we examined word repetition (Test 9 from PALPA, Psycholinguistic Assessments of Language Processing in Aphasia; Kay *et al*., 1992) and words per minute on the Cookie Theft picture description task (BDAE; Goodglass and Kaplan, 1983). Four patients showed severe impairment of repetition, while one had milder impairment. Three of these four individuals were also unable to produce speech in the Cookie Theft picture description task, and three additional cases showed reduced speech fluency. Digit-span was impaired in six patients. We assessed executive function and non-verbal reasoning with Raven’s progressive coloured matrices (Raven, 1962) and the Brixton rule attainment test (Burgess & Shallice, 1997). Eight of the group showed deficits on at least one of these assessments, in line with previous studies which found that deregulated semantic cognition correlated with executive dysfunction in stroke aphasia (Jefferies and Lambon Ralph, 2006; Noonan et al., 2010; Thompson et al., 2018).

### 4.2 Cambridge semantic battery

This assesses semantic retrieval for a set of 64 items across tasks (Adlam, Patterson, Bozeat, & Hodges, 2010; Bozeat, Lambon Ralph, Patterson, Garrard, & Hodges, 2000), including picture naming, word-picture matching, verbal and pictorial semantic associations (Camel and Cactus Test). Word-picture matching involved an array of ten semantically-related items, while the association judgements required a probe to be matched with one of four response options, presented as either pictures or words (in written form and also spoken aloud by the researcher). In line with their varying language output impairment, patients showed large variability during picture naming [percentage correct M(SD) = 58% (40.3)]. In contrast, performance was uniformly at ceiling in word-picture matching [M(SD) = 95.9% (5.2)]. Performance was poorer on the Camel and Cactus Test, which has higher control demands, and there was no difference across modalities [words M(SD) = 79.4 (15.7); pictures M(SD) = 80.4 (14.5)]. Individual test scores are provided in Table 3. All but one of the patients (DF) showed some impairment on this standard semantic battery.

### 4.3 Tests of semantic control

Four tasks manipulated control demands. All of the patients were below the normal cut-off on both verbal tasks and non-verbal judgements. Individual scores are reported in Table 3.

#### 4.3.1 Ambiguity task

This probed the dominant (MONEY) and subordinate (RIVER) meanings of ambiguous words (e.g., BANK) in a four alternative-forced-choice task (Noonan *et al*., 2010). On some trials, there were sentence cues (e.g., for MONEY, I WENT TO SEE THE BANK MANAGER) or miscues that related to the irrelevant interpretation (e.g., THE BANK WAS SLIPPERY). All the patients were below the normal cut-off in all conditions, showed higher performance in the dominant than subordinate condition, and higher performance following cues than miscues (with the exception of VN and PV who were not tested with cues and miscues). Excluding those two cases, a mixed ANOVA examining the effects of dominance (subordinate and dominant) and cueing (no cue, cue and miscue) by group (patients vs. controls from Noonan *et al*., 2010) showed main effects of dominance [F(1,15) = 80.22, p < .001] and cueing [F(2,30) = 28.32, p < .001] plus interactions for dominance by cueing [F(2,30) = 9.51, p = .001], dominance by group [F(1,15) = 48.35, p < .001] and cueing by group [F(2,30) = 24.25, p < .001]. The three-way interaction [dominance by cueing by group [F(2,30) = 7.77, p = .002] reflected the patients’ greater difficulty with subordinate meanings with no cues or when miscues were provided. A supplementary ANOVA including all cases and omitting the cueing factor showed the same effects of dominance [main effect: F(1,17) = 166.30, p < .001; interaction with group: F(1,17) = 123.23, p < .001].

#### 4.3.2 Object use task

This task required patients to select an object to accomplish a task (e.g., bash a nail into wood), with all items represented as photographs (Corbett et al., 2011). The target was either a canonical tool, normally used to complete the task (e.g., HAMMER), or an alternative non-canonical option (e.g., BRICK), presented among a set of five unsuitable distractors, requiring suppression of the irrelevant yet dominant use of the object. All of the patients (except JI, who was below the normal range for the picture Camel and Cactus Test test) were more impaired at selecting non-canonical targets [canonical M(SD) = 92.4 (7.5) vs. alternative M(SD) = 61.7 (19.4); t(10) = 7.70, p <.001]. As a group, they showed poorer performance for non-canonical targets than controls, who were not asked to select the canonical use due to ceiling effects: t(10.6) = 5.99, p < .001 (control data from Corbett et al. 2011).

#### 4.3.3 Synonym tasks

(i) Frequency effects in 96-item synonym judgement (Jefferies *et al*., 2009): In this task, administered to all patients but VN, a probe word was presented with three response options. The words on each trial varied in lexical frequency and imageability (full task details in Jefferies *et al*., 2009). Patients with semantic aphasia, in common with those with “access” impairment, typically do not show sensitivity to frequency (Hoffman, Rogers, & Ralph, 2011; Jefferies, Baker, Doran, & Ralph, 2007; Thompson et al., 2015; Warrington & Cipolotti, 1996), unlike semantic dementia patients with “storage” impairment (Jefferies et al., 2009). The majority of patients (six) showed no frequency effect. Three patients out of eleven showed slightly higher performance during high frequency trials (KQ, CX, DF); one patient (LA) performed better for low frequency trials. We compared our SA sample with the SD patients from Jefferies et al. (2009). ANOVA revealed a frequency by group interaction [F(1,20) = 35.46, p <.001] as well as the main effect of frequency [F(1,20) = 45.84, p < .001]. The SA patients showed no difference between high frequency trials [percentage correct M(SD) = 75.8(10.1)] and low frequency trials [M(SD) = 74.8(11)], unlike the SD patients.
(ii) 84-item synonym judgment task with strong and weak distractors (Noonan et al., 2010; Samson, Connolly, & Humphreys, 2007): Synonyms were presented alongside strong and weak associates as distractors; e.g., dot with point [target], presented with dash [strong distractor] or leg [weak distractor], although VN was not tested. Performance was below the normal cut-off for all patients in trials with strong distractors. With one exception (WB), all patients showed poorer performance when semantic distractors were available. ANOVA looking at distractor strength (related vs. unrelated) and group (patients vs. controls, with control data from Samson et al., 2007) revealed that the patients were more impaired than controls by semantically-related distractors [main effect of distractor strength: F(1,16) = 6.29, p = .023 and distractor strength by group interaction: F(1,16) = 14.22, p = .002].

In summary, we selected patients with multimodal semantic deficits following left hemisphere stroke to take part in this study, since previous work has shown that patients with this profile have deregulated semantic cognition typically associated with damage to key regions implicated in semantic control, particular left inferior frontal gyrus (e.g., Jefferies et al., 2006; Noonan et al., 2010). All eleven patients in this investigation had damage to this region, which is causally implicated in the control of semantic retrieval in healthy participants by inhibitory transcranial magnetic stimulation (e.g. Whitney et al., 2011). In contrast, ventral ATL which is implicated in heteromodal semantic representation by patients with semantic dementia, was intact: this watershed site is rarely damaged in stroke patients (e.g., Payabvash et al., 2011). Consequently, patients with heteromodal semantic deficits following left hemisphere stroke are thought to have semantic ‘access’ deficits that disrupt the ability to flexibly retrieve relevant information to suit the current goals or context. In line with expectations for semantic control impairment, the SA patients in our study were impaired at retrieving non-dominant aspects of meaning across verbal and non-verbal tasks, like previous samples (Corbett et al., 2011; Jefferies & Lambon Ralph, 2006; Noonan et al., 2010). This pattern was seen near-universally, even in patient VN, for whom we had limited data. The patients showed attenuated effects of word frequency on the synonym judgement task compared with a sample of semantic dementia patients examined previously, in line with the profile for semantic ‘access’ deficits. They also showed strong sensitivity to manipulations of semantic control. Difficulties in retrieving weak and non-dominant aspects of knowledge could reflect either loss of this knowledge or difficulties in constraining the retrieval to suit the circumstances. In this context, it is notable that the SA patients were vulnerable to miscuing effects, since it not trivial to explain how these could arise in the absence of control impairment.

A composite score reflecting each patient’s overall semantic control abilities was derived from the Camel and Cactus Tests, Object use and the Ambiguity task without cues (i.e. the semantic control tests that were administered to all participants) using factor analysis. Patients are ordered by this composite score in all the graphs and tables.

## 5 Training study overview

**Figure 2:**
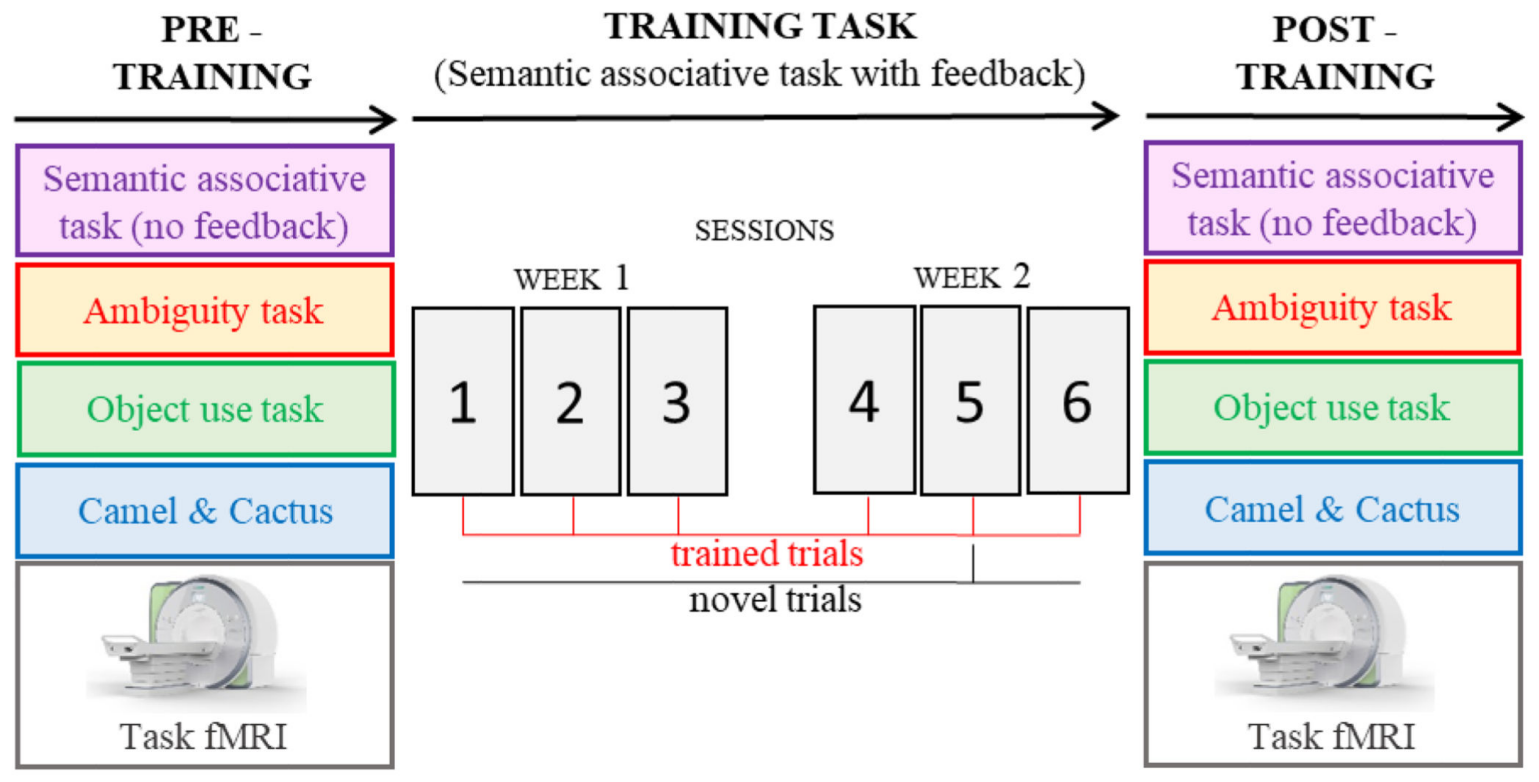
Schematic of study design. Trained trials were repeated in every training session, whereas novel trials were only presented once.

The experimental design is summarized in Figure 2. Patients were trained using a semantic associative task (hereinafter referred to as “training task”), administered in six consecutive sessions across two weeks. We examined training effects by looking at performance (i) over the course of training and (ii) on a semantic associative task that had the same design as the training task but without feedback – administered before and after training. In both cases, generalization was examined by looking at performance on novel trials (i.e. presented only once over the course of training) as opposed to repeatedly trained trials. (iii) We also repeated the ambiguity task, the object use task and the harder trials from the picture Camel and Cactus Test, shortly after the training period, to assess generalization beyond the training paradigm. All eleven patients took part in the behavioral tasks (i.e. training task, semantic associative task with no feedback, ambiguity task, object use task and camel & cactus) with the exception of VN who withdrew from the study and was not tested on the ambiguity and object use tasks after training.

## 6 Behavioural methods

### 6.1 Training task: Procedure

**Figure 3:**
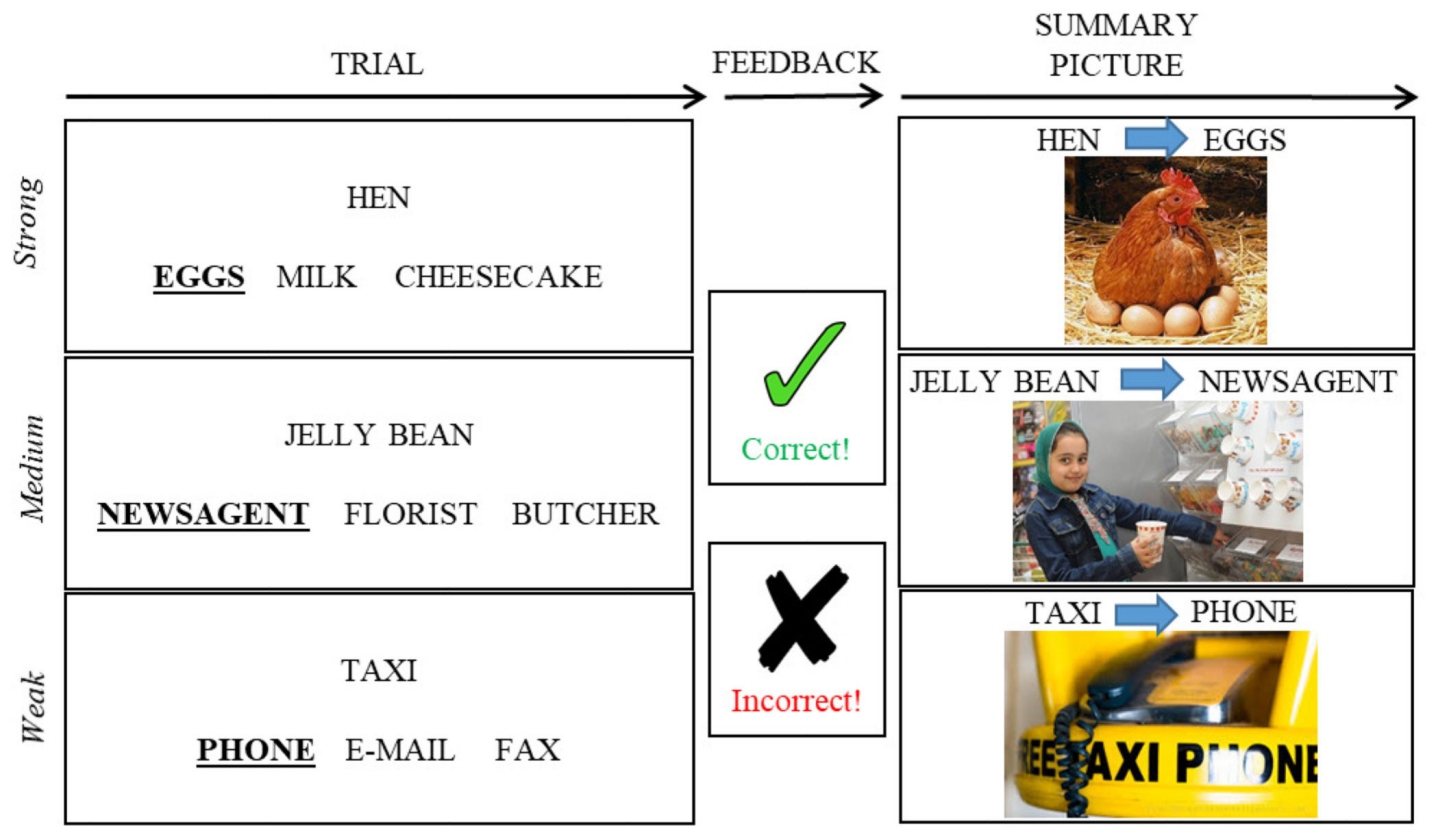
Schematic of training task

Participants performed a three forced-choice semantic association task (see Figure 3). Three words appeared on the bottom of the screen for 2500 ms, during which time they were read out aloud by the examiner, followed by a single probe word appearing at the top. Participants were required to point to one of the three words that had the closest semantic association with the probe word. There was no maximum time allowed for a response; participants were asked to guess if they were unsure. The examiner repeated the words again at the participants’ request, in order to reduce the impact of reading impairment on performance.

At the end of each trial, participants were provided with feedback as to whether they were correct or incorrect. This took the form of a green tick with the word “correct”, or a red cross with “incorrect”. An image that reinforced the relevant semantic association was also displayed together with the probe and the correct response. For example, for the association between TAXI and PHONE, an image of a taxi free phone was displayed (see Figure 3). A verbal description was added to summarise the link between the target and probe if the picture was unclear to the patient. These images were presented for both correct and incorrect trials. The feedback and summary picture were presented until the patient was ready to move onto the next trial. Trials were separated by 250 ms fixation cross.

We manipulated the strength of association between the probe and target. Strong associations required little control over retrieval, since the dominant association for the probe corresponded to the target, while medium and weaker associations required more control over semantic activation in order to focus retrieval on the relevant relationship and suppress stronger but currently irrelevant associations (cf. Badre and Wagner, 2005; Whitney *et al*., 2011). The distractor words for each trial were related to the target to increase inhibitory demands. For example: TAXI – PHONE not E-MAIL, FAX (weak association); JELLY BEAN – NEWSAGENT not FLORIST, BUTCHER (medium-strength association); HEN – EGGS not MILK, CHEESECAKE (strong association, see Figure 3). Forty trials were repeated in every session, whereas 25 novel trials were presented to test for generalisation (see Figure 2). This gave 65 trials per session for analyses. Each training session started with 3 practice trials which were omitted from the analysis and lasted around 15-20 minutes. The order of the training sessions was counterbalanced across participants. The strength of association for each of these trials was matched across sessions (i.e. each session had the same overall level of difficulty). Associative strength was derived from Edinburgh Association Thesaurus (EAT; Kiss *et al*., 1973). Approximately one third of the trials in both the repeated and novel conditions were strong, medium and weak associations. For the repeated trials, the average association on the EAT was as follows: Strong M (SD) = 5.9 (0.3); Medium M (SD) = 4.8 (0.4); Weak M (SD) = 3.1 (0.5). For the novel trials, the average association on the EAT was similar for strong, medium and weak trials: Strong M (SD) = 6.1 (0.4); Medium M (SD) = 4.8 (0.5); Weak M (SD) = 3.2 (0.6).

The six training sessions were conducted over a 2-3-week period, with sessions separated by at least 24 hours. This was motivated by accumulating evidence that brief intensive aphasia therapies are associated with better outcomes than more distributed and prolonged interventions (Stahl et al., 2017). The task was presented using E-Prime 2.0 (Psychology Software Tools). The complete list of stimuli is provided in the Appendix, Table 1.

### 6.2 Semantic associations without feedback: Procedure

Before and after training, participants performed a task with the same format as the training task, but without the provision of feedback and the linking picture after each trial. As in the training task, associative strength between the probe and target was manipulated; this was matched across the pre- and post-training sessions. There were 82 trials: 24 were trained (16 of these were trained repeatedly, and 8 were trained only once; all trained trials were tested in both pre- and post-training sessions) and 58 were not (34 of these trials were repeatedly tested in both pre- and post-training sessions whereas 24 were tested either prior or after training). The complete list of trials is provided in the Appendix, Table 2. This procedure therefore assessed whether (i) there was an overall improvement in selecting the correct semantic associate among distractor following training and (ii) whether any improvement was restricted to trials that had been trained, or generalised to trials that had not been trained.

### 6.3 Untrained semantic tasks: Procedure

A set of semantic assessments were repeated in the two weeks before and after training, to characterise any changes in performance over the training period. After training we retested the ambiguity task (dominant vs. subordinate without cues), the object-use task and a subset of 26 of the harder Camel and Cactus Test trials (these trials were selected according to the performance of an earlier sample of SA cases who had completed the full assessment; they were the items with the poorest performance, across both picture and word versions). Individual analyses were performed on overall accuracy (without distinguishing between conditions) to retain sufficient statistical power.

### 6.4 Behavioural analyses overview

Repeated-measures ANOVAs and 2-tailed paired samples t-tests were used to assess training effects and experimental manipulations (e.g. trained vs. novel, associative semantic strength) at the group level. Individual performance was analysed using McNemar tests when the same trials were tested at different time points (such as for repeatedly trained trials during the first vs. last session of the training task). When different trials were presented before and after training, such as for novel trials of the training task, chi-square and Fisher’s exact tests were used.

## 7 Behavioural results

### 7.1 Training task: Results

#### Group level effects during training task

**Figure 4:**
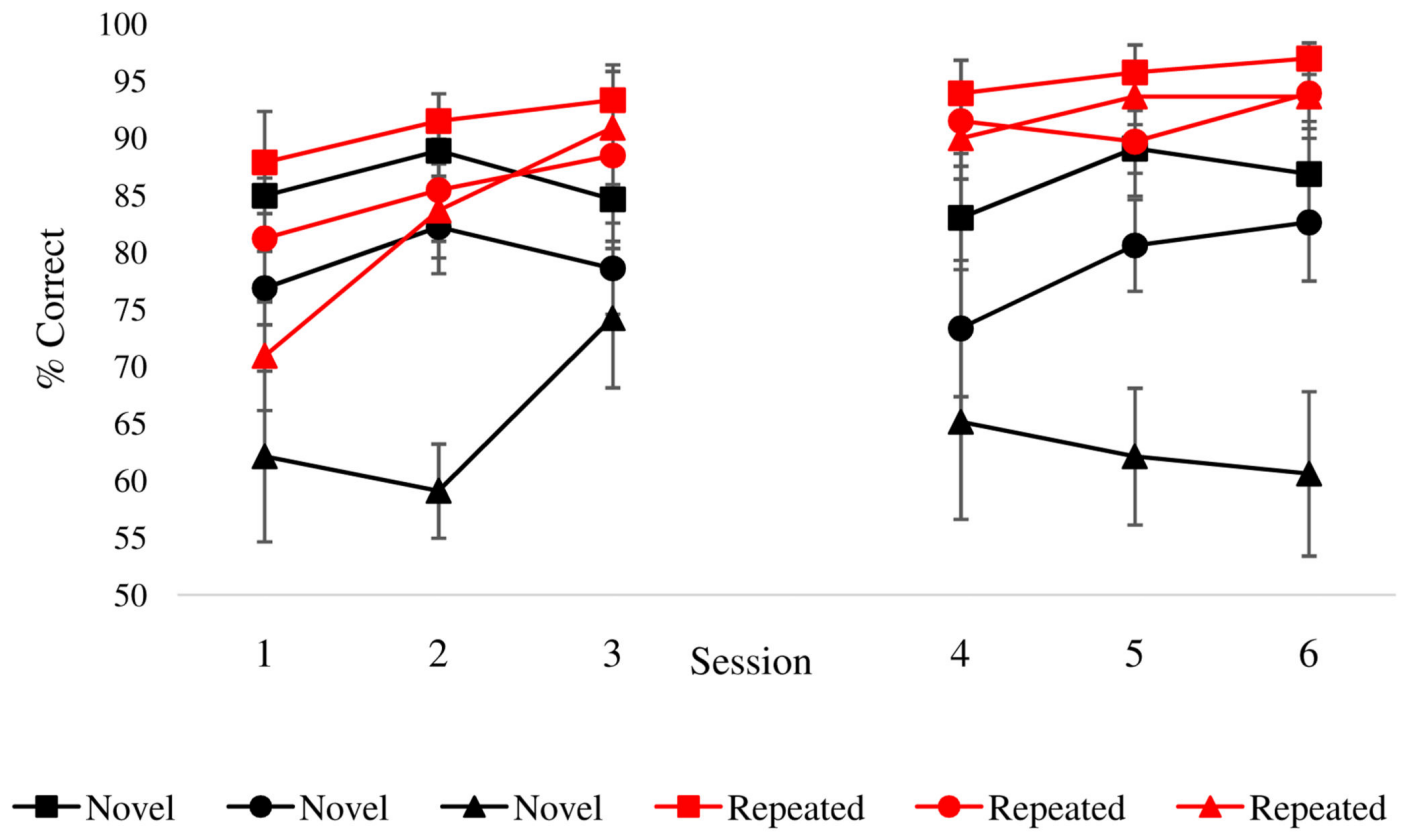
Training task, group level analysis: sessions by repetition (repeated, novel) by associative strength (strong, medium, weak). Error bars show SEM.

**Figure 5:**
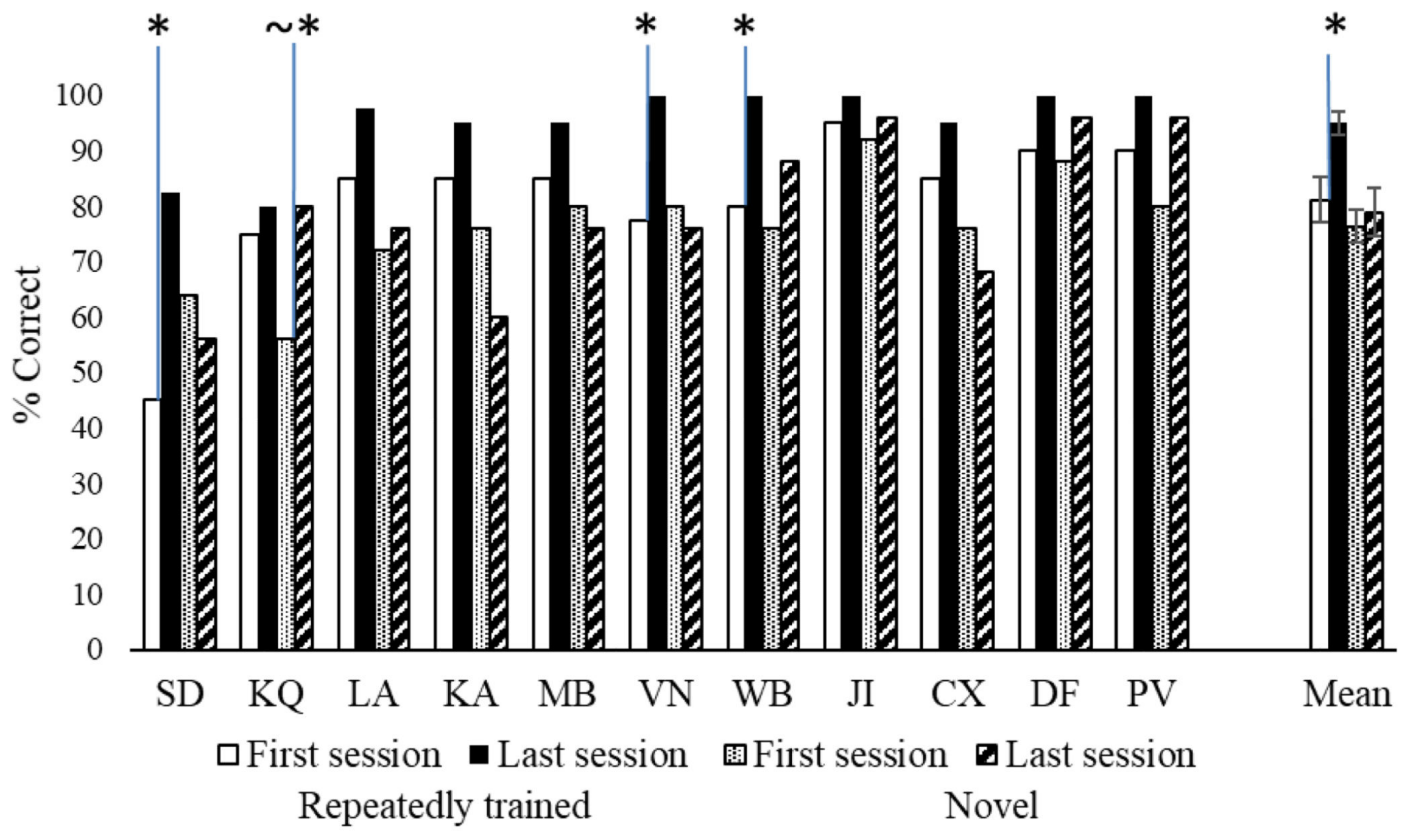
Training task, individual analysis: sessions (first vs. last) by repetition (repeated, novel). * = significant (p < .05) difference between conditions; ~* = difference between conditions approaching significance (p ≤ .07). Error bars show SEM.

Figure 4 shows the key results. A 6 (training sessions) by 2 (repeated vs. novel) by 3 (strong, medium and weak associations) ANOVA revealed an overall improvement across sessions [main effect of training session [F(5,50) = 4.1, p = .004] and higher accuracy for repeated as opposed to novel items [F(1,10) = 68.61, p < .001]. There was also a main effect of strength of association [F(2,20) = 32.57, p <.001], revealing higher accuracy for strong vs. medium vs. weak associations. There were also two interactions. There was a stronger training effect for repeated trials [training session by repetition: F(5,50) = 3.01, p = .018]. Follow-up tests comparing the first and last sessions showed that the trained items increased in accuracy [t(10) = 4.84, p = .001], while novel items did not [t(10) = .709, p = .494, Figure 5]. In addition, repetition interacted with strength of association [F(2,20) = 11.01, p =.001]. Post-hoc comparisons of accuracy by associative strength across all training sessions revealed a bigger effect of strength of association for novel [strong vs. weak: t(10) = 5.78, p = .001 and medium vs. weak: t(10) = 5.15, p = .002, Bonferroni corrected for six comparisons] as opposed to repeated trials [strong vs. weak: t (10) = 3.5, p = .036; Bonferroni corrected for six comparisons; all the other comparisons were non-significant]. All other interactions were non-significant [F < 1.5].

#### Individual analysis during training task

Figure 5 shows key results. Effects of repetition and strength of association were examined in each individual patient using separate analyses to increase statistical power. For repeated trials, 3 patients (SD, WB and VN) showed significant improvement from session 1 vs. session 6 [McNemar p ≤ .008]. In all the other cases performance was higher in the last vs. first session of training, but this did not reach significance. For novel trials, only KQ showed a trend towards higher accuracy in the last vs. first session [χ^2^ (1) = 3.31, p = .069]. SD, KQ and PV showed increased accuracy between first and last session for, respectively, strong [χ^2^(1) = 4.75, p = .029], medium [χ^2^(1) = 4.75, p = .029] and low [Fisher’s exact test: p = .037] strength of association trials. No significant improvement was found for the all other patients [χ^2^ (1) < 3].

In summary, out of 22 sets of items (11 patients by novel/repeated sets), only 3 showed a statistically significant effect of the training, although 16 sets showed a numerical difference in the correct direction.

### 7.2 Semantic associations without feedback: Results

#### Group level effects comparing pre- and post-training sessions

**Figure 6:**
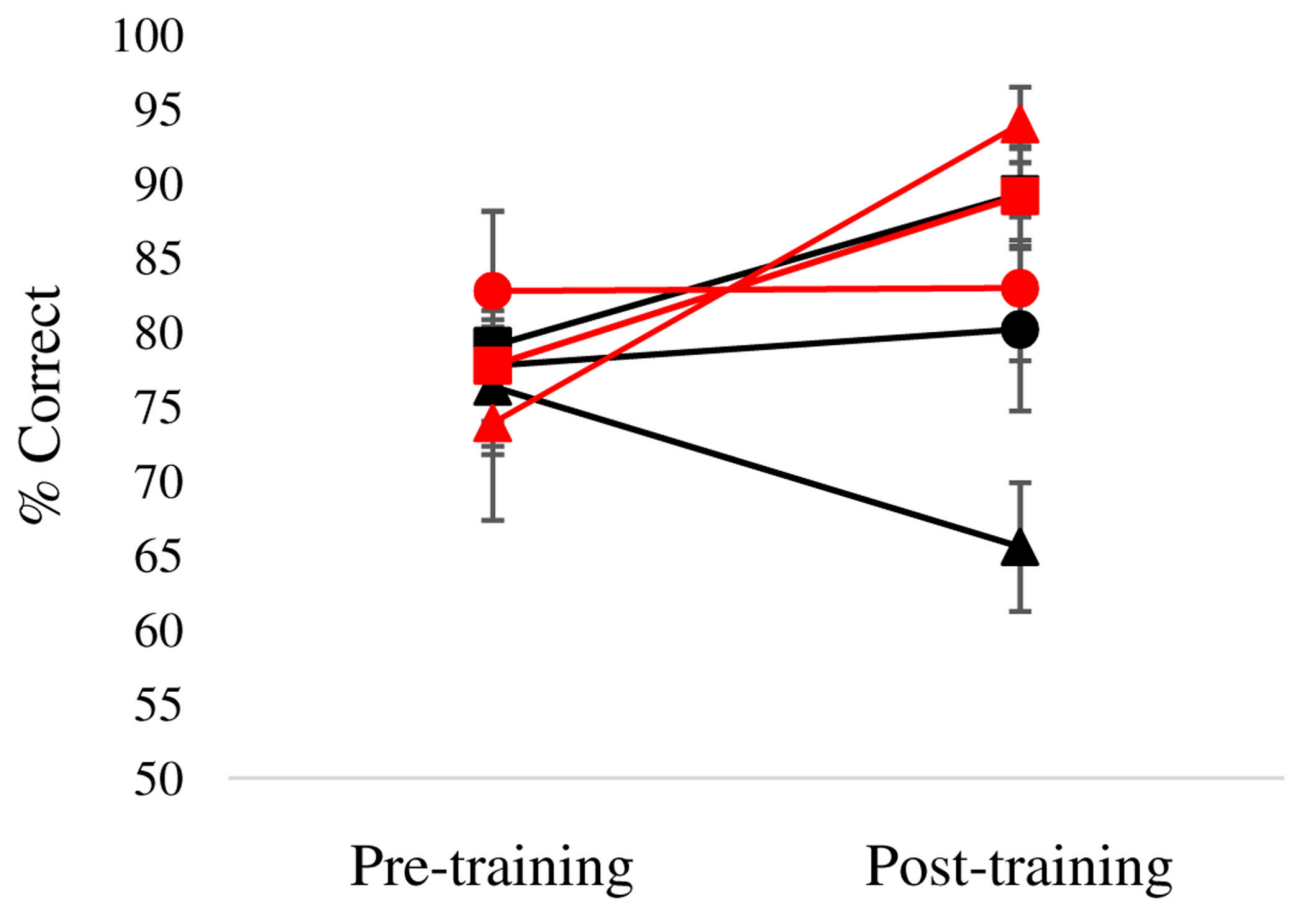
Semantic associative task without feedback, group level analysis: session (pre vs. post) by training (trained, untrained) by associative strength (strong, medium, weak). Error bars show SEM.

Figure 6 shows key results. A 2 (session: pre vs. post) by 2 (trained vs. untrained) by 3 (strong, medium and weak associations) ANOVA revealed main effects of session [F(1,10) = 17.61, p = .002], training [F(1,10) = 13.7, p = .004] and strength of association [F(2,20) = 3.75, p = .041]. An interaction of session by training [F(1,10) = 14.16, p = .004] reflected greater improvement for trained trials. Paired t-tests showed that there was an improvement in accuracy on trained items [t(10) = 3.65, p = .008, Bonferroni corrected for two comparisons] but no significant improvement on untrained items [t(10) = 1.56, p = .3, Bonferroni corrected for two comparisons]. There were also interactions of training by strength of association [F(2,20) = 4.64, p = .022] and a three-way interaction [training by strength of association by session: F(2,20) = 8.94, p = .002]. This revealed that for trained trials, there was a trend toward improvement after training for weak trials only [t(10) = 3.01, p = .072, Bonferroni corrected for six comparisons, all the other comparisons were non-significant]. For untrained trials, performance improved for strong trials only [t(10) = 3.17, p = .06, Bonferroni corrected for six comparisons]; no difference was found for medium associative strength and performance dropped for weak associative trials [but this was not significant: p = .33].

#### Individual analysis comparing pre- and post-training sessions

**Figure 7:**
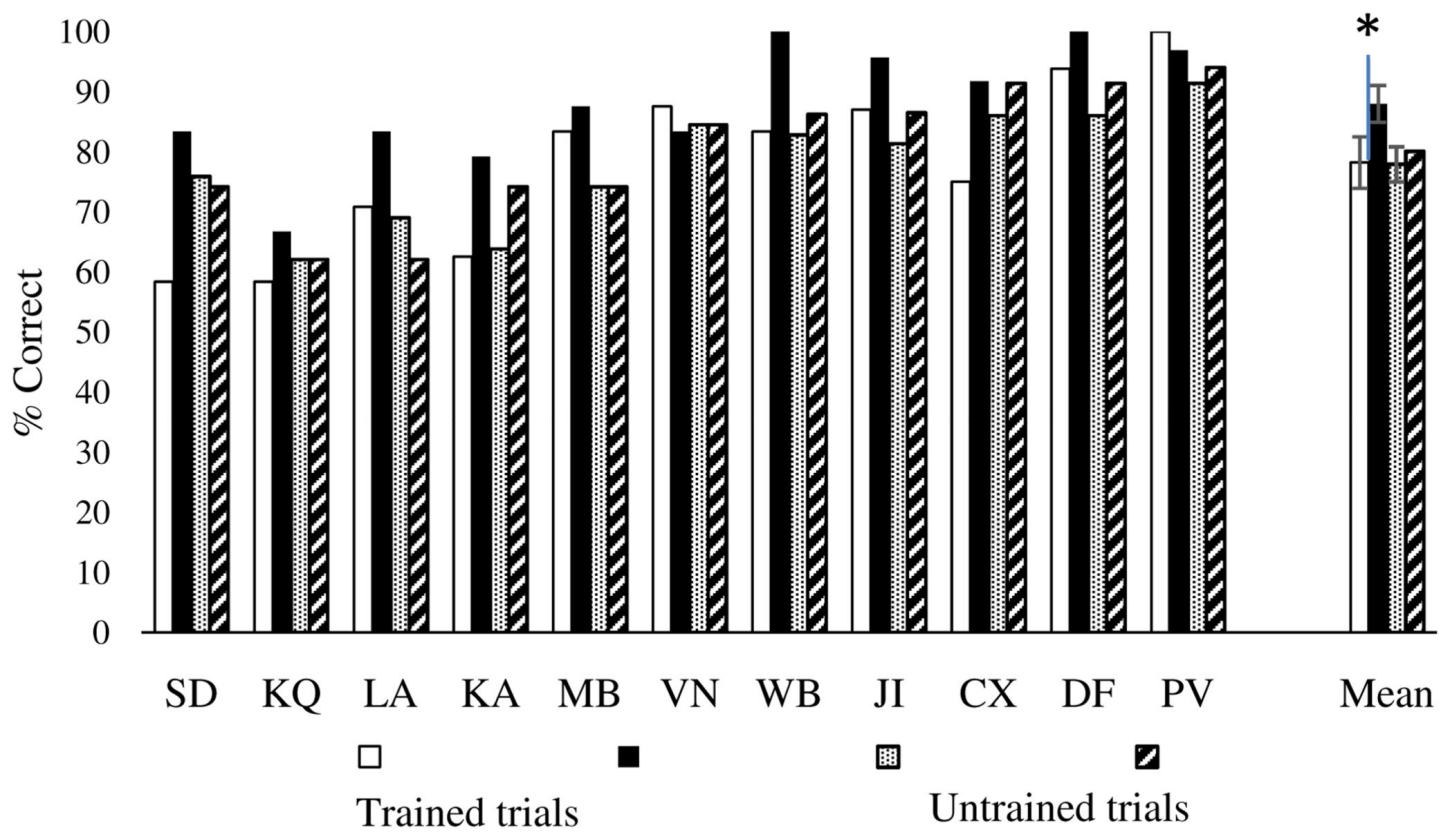
Semantic associative task without feedback, individual analysis: session (pre vs. post) by training (trained, untrained). Error bars show SEM.

Effects of training and strength of association were examined in each individual patient using separate analyses to increase statistical power. Training effects were examined using McNemar for trials tested before and after training and Chi-square for trials tested either in pre- or post-training session. None of the patients showed a significant improvement in accuracy for untrained [McNemar p ≥ .125] or trained trials [McNemar p ≥ .125, see Figure 7]. Similarly, none of the patients showed an improvement for untrained and non-repeated trials [i.e. not tested before and after training, χ^2^ (1) < 2]. KQ, JI, MB and WB showed an increase in accuracy after training for high strength of association trials [respectively: χ^2^ (1) = 4.31, p = .038; Fisher’s exact test: p = .026; χ^2^ (1) = 3.72, p = .054: Fisher’s exact test: p = .005]. No significant improvement was found for medium and low trials for the all the patients [χ^2^ (1) < 2].

### 7.3 Untrained semantic tasks: Results comparing pre- and post-training sessions

**Figure 8:**
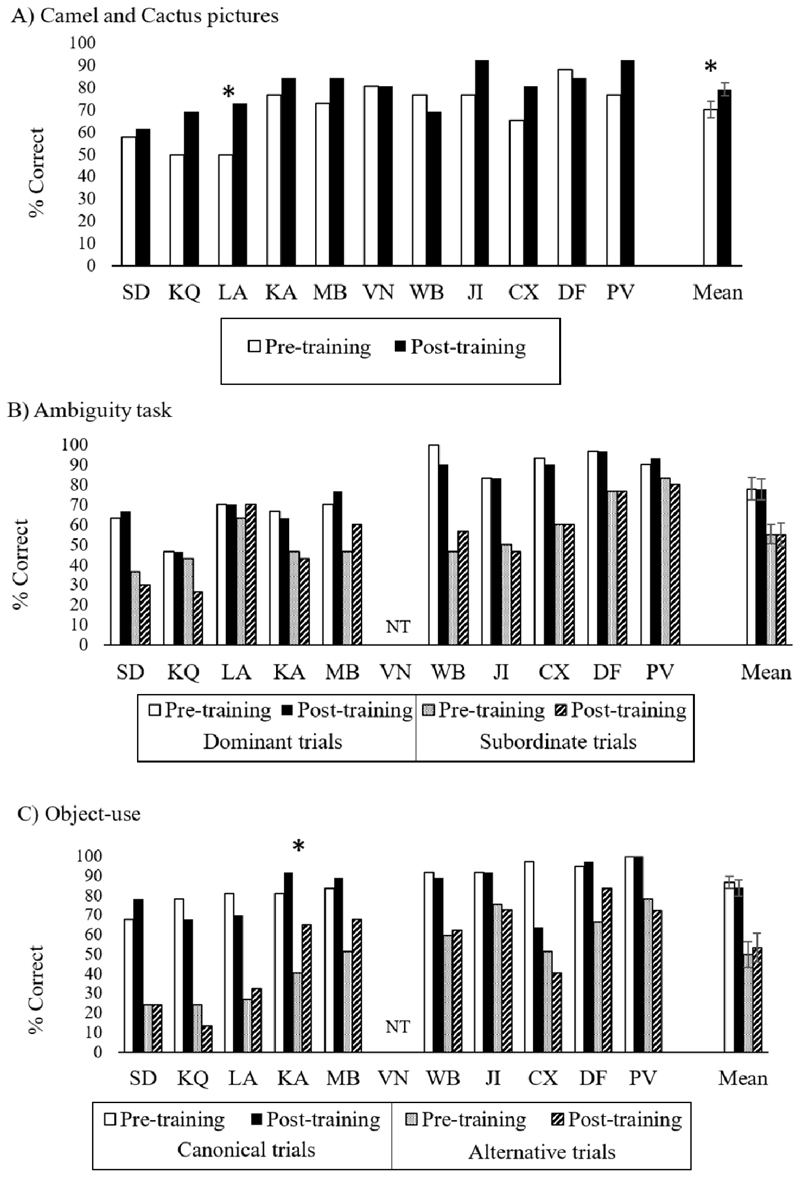
Accuracy in semantic control tasks. NT = not tested; * = significant (p < .05) improvement after training. Error bars show SEM.

Figure 8 shows key results.

(i) The 26-item Camel and Cactus Test showed a significant increase in accuracy after training [t(10) = 3.04, p = .012; pre-training M(SD) = 70.3 (12.8); post-training M(SD) = 79.4 (9.9)]. This suggests that the group did show some generalisation of the training. Individual analyses showed that only patient LA significantly improved after training [McNemar: 4.17, p = .041].
(ii) For the ambiguity task, a 2-by-2 ANOVA looking at the effect of time (pre vs. post training) and dominance (dominant vs. subordinate meaning) on accuracy revealed no change over time [F < 1], lower accuracy for subordinate trials [main effect of dominance: F(1,9) = 32.61, p < .001] and no interaction [F < 1]. None of the patients showed a significant improvement pre vs. post accuracy [McNemar p > .180].
(iii) The object use task also showed no change from pre- to post-training sessions [main effect of time: F < 1] and better accuracy for canonical vs. alternative use trials [F (1,9) = 74.29, p < .001] with no interaction [F < 1]. One patient (KA) showed higher accuracy after training [McNemar: 5.50, p = .019].

## 8 Discussion

In a group of semantic aphasia (SA) patients with multimodal semantic deficits stemming from poor semantic control, we assessed the effects of a training task designed to encourage flexibility in the retrieval of semantic associations. We found improvement in the accessibility of semantic associations; however, this effect was more marked for items that were trained repeatedly. There was little evidence of generalisation of this training effect to novel items in the training task itself. Nevertheless, the group showed some generalisation because a similar yet untrained task involving the retrieval of semantic associations (Camel & Cactus Test) also improved with training. It is not clear why the novel items within the training task and the Camel & Cactus Test showed different patterns; one possibility is that the harder Camel and Cactus items were highly sensitive to changes in semantic control.

Training often does not generalise to new items – for example, in picture naming therapies for participants with aphasia, often only the trained item set shows facilitation (Davis & Pring, 1991; Marshall et al., 1990; Pring et al., 1993). Training effects also typically fail to generalise to untrained tasks – for example, following protocols to increase cognitive control or working memory capacity, performance gains often do not extend to untrained paradigms that recruit the same putative cognitive processes (Melby-Lervåg & Hulme, 2013). Our results are broadly consistent with this pattern of weak or non-existent generalisation. However, the group-level effect on one of our background semantic tasks (Camel & Cactus Test) is promising, suggesting this type of semantic training might be more broadly beneficial. There is also some evidence that some individuals in our case-series benefitted more than others: although our single-subject analyses had substantially-reduced statistical power relative to the group-level analyses, we found that one individual case showed an effect of the semantic training task on novel trials, while three individuals showed significant changes on trained trials. More research is needed to predict which patients are most likely to show benefits from semantic training: unlike studies of Semantic Feature Analysis (e.g., Boyle, 2010), there is no suggestion in our data that the most impaired patients were least able to benefit, even though these more severely affected individuals are likely to have had additional deficits in cognitive control Jefferies & Lambon Ralph, 2006).

There are relatively few studies of semantic rehabilitation which use semantic judgements as opposed to lexical retrieval (e.g. picture naming tasks) as the outcome measure (see Efstratiadou et al. 2018, for a review). Given that semantic deficits are common in aphasia, an important aspect of the current investigation is our demonstration that single-item semantic judgements (including non-verbal semantic decisions) show improvement following semantic training, at least at the group level. Moreover, since multimodal semantic deficits in aphasia are associated with specific difficulties in retrieving non-dominant aspects of knowledge, our study provides an example of how training on a task designed to tap this particular difficulty can lead to improvements in performance in patients with SA. It might be that more intensive training over a longer period, with more sessions, or more trials per session, could produce a larger effect. In addition, it might be possible to optimise the training to encourage relevant patterns of retrieval across different contexts. For example, the word bank could be trained on associations of its dominant (i.e. financial institution; e.g. bank – money, not morning, heart or child) or subordinate (i.e. edge of a lake/river; e.g. bank – river, not dress song or birth) meanings on consecutive trials. This could promote flexible retrieval of conceptual knowledge according to the task requirement. The current investigation is also insufficient to identify the underlying cognitive change that was responsible for the improved performance that we saw: the training task may have facilitated the recovery of semantic control processes, for example through additional recruitment in undamaged parts of the semantic control network (cf. Hallam et al., 2018), or it may have allowed patients to identify compensatory strategies beyond semantic control.

In conclusion, patients with semantic control deficits may benefit from training tasks that encourage the retrieval of diverse types of semantic associations. There were clear individual differences in our sample, suggesting not all patients will be able to generalise the effects of training to untrained items or tasks. Our results confirm the need to develop more effective training protocols that target semantic control and executive processes in patients with aphasia, since this kind of training is thought to be more likely to produce functionally-meaningful improvement (Vas *et al*., 2016; Han *et al*., 2018). This may be the case especially in people with semantic aphasia who have difficulty regulating their retrieval of conceptual information, yet little loss of semantic knowledge from long-term memory. Not enough is yet known about the optimisation of such cognitive training – for example, too much repetition of the same items might reduce mental flexibility, as these items become too dominant within the mental landscape. However, too little repetition might reduce the opportunity for patients to accurately retrieve diverse types of associations for themselves, and thereby acquire more effective retrieval strategies. Moreover, there is a need for research that confirms whether different approaches to neurorehabilitation are maximally effective in patients with semantic deficits that have different underlying causes, such as contrasting patients with deregulated semantic retrieval following left hemisphere stroke with patients with degraded conceptual knowledge in the context of semantic dementia.

## Figures and Tables

**Table 1:** Demographics, non-semantic background task and aphasia classification

Test	Max	Cut-off	SD	KQ	LA	KA	MB	VN	WB	JI	CX	DF	PV
Age			61	78	60	67	58	48	66	59	78	40	57
Sex			F	M	F	M	M	F	M	F	F	F	F
Years since CVA			7	7	9	24	14	4	7	7	5	8	8
Non-semantic background tasks													
Category fluency (mean per cat.)	-	8	**0**	**7**	**5**	**4**	**7**	**0**	**0**	14	17	17	15
Cookie theft (words/minute)	-	-	0	18	9	12	37	0	0	60	54	37	38
PALPA 9, real word repetition	16	14	**0**	14	**12**	15	16	**0**	**2**	16	15	16	**6**
Forward digit span	-	5	**0**	**4**	**2**	5	**4**	**0**	**0**	6	5	5	6
Raven’s coloured matrices	36	28	31	29	31	**24**	30	32	34	**19**	**21**	33	33
Brixton (correct)	54	28	**21**	**7**	**18**	**26**	**23**	**6**	31	**24**	31	30	39

*Note*: Scores are number of correct, unless specified. CVA: Cerebrovascular accident. NT = unavailable for testing, Bold underlined numbers denotes impaired scores (below cut-off).

**Table 2:** Patients’ lesion analysis

				Fronto-lateral					Parieto - temporal									
Patient ID	Lesion size^*^	SMA/PMC	FP		DLPFC	ant-IFG	mid-IFG	post-IFG	SMG	AnG	pMTG	STG	MTG	ITG	FuG	TP	PHG	Hpc
									Brodmann Areas									
		6	10	9	46	47	45	44	40	39	37	22	21	20	36	38	28	28
SD	12	1	-	1	1		1	1	2	1	1	2	-	-	-	1	-	-
KQ	15	2	-	2	-	2	2	2	1	-	-	2	-	-	-	-	-	-
LA	15	2	-	2	-	2	1	2	2	1	2	2	2	1	-	-	-	-
KA	8	2	-	-	-	-	-	1	1	-	2	-	-	-	-	-	-	-
MB	7	1	-	-	-	-	1	2	1	1	1	-	-	-	-	-	-	-
VN	17	2	-	1	1	2	2	2	2	2	-	2	1	-	-	1	-	-
WB	14	2	-	-	-	2	1	2	1	1	1	2	1	-	-	-	-	-
JI	15	2	-	-	-	-	-	2	2	1	1	-	-	-	-	-	-	-
CX	4	1	-	-	-	-	-	1	-	-	1	1	1	-	-	-	-	-
DF	9	-	-	-	-	-	1	2	-	-	-	2	-	-	-	-	-	-
PV	14	2	-	-	1	-	2	2	2	-	-	2	-	-	-	-	-	-

*Note*. MRI scans were manually traced onto Damasio templates. Lesion size* was calculated as % template damaged. For areas not comprehensively characterized by Damasio templates, analyses were combined with manual analysis of the structural scan with the help of a trained radiographer. Quantification of lesion: 2 = complete destruction/serious damage to cortical grey matter; 1 = partial destruction/mild damage to cortical grey matter; “-“ = intact. Anatomical abbreviations: SMA/PMC: Supplementary Motor Area/ Premotor Cortex; FP: Frontal Pole; DLPFC: Dorsolateral Prefrontal Cortex; ant-IFG: Inferior Frontal Gyrus, pars orbitalis; mid-IFG: Inferior Frontal Gyrus, pars triangularis; post-IFG: Inferior Frontal Gyrus, pars opercularis; SMG: Supramarginal Gyrus; AnG: Angular Gyrus; pMTG: posterior Middle Temporal Gyrus; STG: Superior Temporal Gyrus; MTG: Middle Temporal Gyrus; ITG: Inferior Temporal Gyrus; FuG: Fusiform Gyrus; TP: Temporal Pole; PHG: Parahippocampal Gyrus; Hpc: Hippocampus.

**Table 3:** Background semantic tasks: individual scores

Semantic background tasks													
Test	Max	Cut-off	SD	KQ	LA	KA	MB	VN	WB	JI	CX	DF	PV
*Cambridge Semantic Battery*												
Picture naming	64	59	**1**	61	**19**	**50**	**50**	**0**	**3**	60	**56**	62	**46**
Word-Picture matching	64	62	63	62	**60**	64	62	**61**	**52**	62	64	62	63
Word CCT	64	56	**39**	**43**	**29**	**53**	**52**	**50**	57	59	61	60	56
Picture CCT	64	52	**31**	**44**	**45**	56	57	59	54	**45**	53	61	61
*Ambiguity task*													
Miscued dominant	30	30	**12**	**13**	**13**	**14**	**19**	NT	**21**	**20**	**24**	**26**	NT
Miscued subordinate	30	28	**7**	**10**	**14**	**8**	**15**	NT	**18**	**10**	**18**	**19**	NT
No cue dominant	30	28	**22**	**18**	**24**	**22**	**26**	**27**	**27**	**24**	**28**	**28**	**27**
No cue subordinate	30	28	**11**	**9**	**14**	**14**	**17**	**17**	**19**	**19**	**21**	**19**	**21**
Cued dominant	30	30	**23**	**21**	**19**	**22**	**23**	NT	**23**	**24**	**27**	**29**	NT
Cued subordinate	30	29	**25**	**14**	**20**	**18**	**28**	NT	**24**	**19**	**23**	**25**	NT
*Synonym task (96 items)*													
High frequency	48	42	**27**	**33**	**32**	**37**	**38**	NT	**39**	**34**	**41**	43	**40**
Low frequency	48	44	**30**	**25**	**39**	**37**	**38**	NT	**42**	**32**	**37**	**38**	**41**
*Synonym task with distractors (84 items)*												
Strong	42	40	**15**	**12**	**13**	**20**	**23**	NT	**30**	**21**	**22**	**17**	**38**
Weak	42	35	**25**	**23**	**29**	**24**	**30**	NT	**31**	**27**	**28**	**39**	36
*Object use*													
Alternative	37	34	**14**	**13**	**14**	**21**	**22**	**24**	**22**	34	**26**	**29**	**32**
Canonical	37	-	32	31	29	35	35	33	33	37	37	37	*37*

*Note*: Scores are number of correct, unless specified. Legend: NT = unavailable for testing. Bold underlined numbers denote impaired scores (below cut-off). Control data were not collected for the canonical object use task; since controls performed close to ceiling on the harder alternative use version of this test.
